# Effective radiation dose in radiostereometric analysis of the hip

**DOI:** 10.1080/17453674.2020.1767443

**Published:** 2020-05-26

**Authors:** Ian F Blom, Lennard A Koster, Bart Ten Brinke, Nina M C Mathijssen

**Affiliations:** a Department of Radiology, Reinier de Graaf Groep, Delft, The Netherlands;; b Department of Orthopaedic Surgery, Leids Universitair Medisch Centrum, Leiden, The Netherlands;; c Department of Orthopaedic Surgery, Reinier de Graaf Groep, Delft, The Netherlands

## Abstract

Background and purpose — Radiostereometric analysis (RSA) is the gold standard to study micromotion of joint replacements. RSA requires the acquisition of additional radiographs increasing the radiation dose of patients included in RSA studies. It is important to keep this dose as low as possible. Effective radiation dose (ED) measurements of RSA radiographs for different joints were done by Teeuwisse et al. some years ago using conventional radiology (CR); for total hip arthroplasty (THA), Teeuwisse et al. reported an ED of 0.150 milliSievert (mSv). With the modern digital radiography (DR) roentgen technique the ED is expected to be less.

Material and methods — In this phantom study, simulating a standard patient, the ED for hip RSA radiographs is determined using DR under a variety of different roentgen techniques. The quality of the RSA radiographs was assessed for feasibility in migration analysis using a (semi-)automatic RSA analysis technique in RSA software.

Results — A roentgen technique of 90 kV and 12.5 mAs with additional 0.2 copper (Cu) + 1 mm aluminum (Al) external tube filters results in an ED of 0.043 mSv and radiographs suitable for analysis in RSA software.

Interpretation — The accumulated ED for a standard patient in a 2-year clinical hip RSA study with 5 follow-up moments and a double acquisition is below the acceptable threshold of 1.0 mSv provided by the EU radiation guideline for studies increasing knowledge for general health.

Since its introduction in 1970 by Göran Selvik (1989), radiostereometric analysis (RSA) has frequently been used to study micromotion of orthopedic implants (Kärrholm et al. 1994, Ryd et al. 1995, Valstar et al. 2002). An RSA study consists of several follow-up moments, each requiring 2 simultaneously taken radiographs, in addition to regular imaging. This results in an increased radiation dose for patients in an RSA study. As stated in the RSA ISO standard, precision of an RSA study needs to be determined with double examinations, adding another RSA radiograph (ISO 16087:2013). In several countries medical ethics committees do not permit the acquisition of double RSA examinations because of the added radiation dose (Valstar et al. 2005).

In general, a combination of increasing kiloVoltage (kV) and decreasing milliAmpere-seconds (mAs) results in a decrease in radiation dose. However, decreasing the radiation dose results in a lower image quality (Bushong 1975, Fauber et al. 2011, Carroll 2014, Ma et al. 2014). Decreasing radiation dose, while the image quality remains acceptable for the purpose, is called the As Low As Reasonably Achievable (ALARA) principle (ICRP 1955). According to Teeuwisse et al. (1998) an RSA radiograph of the hip, using computed radiography (CR) roentgen detectors, has an effective radiation dose (ED) of 0.150 miliSievert (mSv) (Valstar 2001). However, most RSA studies do not provide the ED of the applied roentgen technique and thus the actual radiation dose remains unknown.

Currently, digital radiography (DR) roentgen systems are becoming the standard that provides better image quality with similar or lower radiation dose (Bragdon et al. 2003, Ching et al. 2014).

In addition to DR detectors, modern mobile X-ray tubes contain external tube filters, which can be applied to improve image quality.

In order to assess the dose given to hip patients in an RSA study, we:

1. determined the ED of an RSA radiograph with standard roentgen settings in a hip phantom model using a DR roentgen system with and without external tube filters; and
2. determined the optimal roentgen settings in a hip phantom model using a DR roentgen system.

## ^Material and methods^

### ^RSA set-up^

2 DR system roentgen tubes were used: 1 fixed ceiling tube (DigitalDiagnost, Philips, Best, the Netherlands) and a mobile tube (MobileDiagnost wDR, Philips, Best, the Netherlands). Both tubes were used in combination with a Philips large Skyplate detector of 35**×**43 cm (2,330**×**2,846 pixels, image resolution 165 dpi). Roentgen images were saved as lossless JPGs for analysis in model-based RSA. The tubes were positioned over a carbon calibration cage (Carbonbox number 20, Medis Specials BV, Leiden, the Netherlands) angulated at 20° to the vertical and the roentgen beams were collimated to fit the indicated areas, with sizes of 34**×**41 cm, on the calibration cage. The source–image distance (SID) for this study was 160 cm, which is similar to clinical RSA studies using this type of calibration cage. The object-image distance (OID) is 40 cm and therefore the source–object distance (SOD) is 120 cm.

### ^Effective radiation dose^

**Figure 1.**
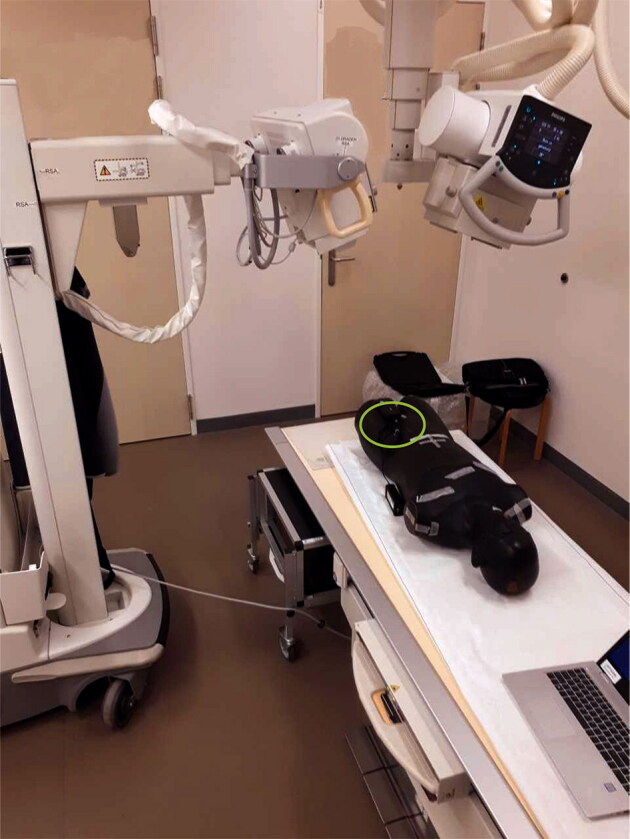
RSA set-up with Alderson phantom and Piranha meter (within the green circle) positioned on top of the phantom to measure the entrance dose. Source–image distance (SID) = 160 cm, object-image distance (OID) is 40 cm and source–object distance (SOD) is 120 cm. Tube angulation is 20° for both tubes.

An Alderson phantom was used to simulate an adult pelvis including soft tissue. A Piranha-meter Multi 657 (RTI, Mölndal, Sweden), with backscatter protection, was positioned in the RSA set-up on top of the Alderson phantom to measure absorbed dose (AD) (Figure 1) (Bushong 1975, Carroll 2014). Based on the measured AD, the ED was calculated for each tube using PCXMC software including all pelvic tissue weighting factors of ICRP publication 103 (STUK, Helsinki, Finland version 2.0.1.4) (ICRP 1991, Veldkamp et al. 2012). The standard roentgen settings as indicated for RSA radiographs of the hip by Valstar were used to determine ED in this study (Valstar 2001). For the medio-lateral tube 73 kV, 25 mAs; latero-medial 90 kV, 12.5 mAs and no external tube filtration. RSA images were also made with the standard roentgen settings and different external tube filters (2 mm Al, 0.1 Cu + 1 mm Al, and 0.2 Cu + 1 mm Al).

### ^Optimal roentgen settings^

In this study we define optimal roentgen settings as the settings where ED is the lowest in combination with RSA images that are of such quality that they can be used for RSA analysis. In order to determine the ED for different roentgen settings, the same set-up was used as to determine the ED for standard settings, but now with a variety of different, but identical for both tubes, roentgen settings (Table 1).

**Table 1.** The different settings for tube voltage (kV), current exposure time product (mAs), and external tube filters, in all possible combinations, that were used to determine ED and image quality

Tube voltage (kV):	77, 85, 90 and 102
Exposure (mAs):	8, 12.5, 16 and 20
External tube filter:	No filtration, 2 mm Al, 0.1 mm Cu + 1 mm Al, and 0.2 mm Cu + 1 mm Al

All the ED measurements, including standard roentgen settings with and without external tube filtering, were performed twice. The largest of the 2 calculated EDs for each combination of roentgen settings is reported. All RSA measurements were performed by 1 author (IB) and calculation of 10% of the RSA measurements, selected by a random number generator, was performed by a second author (LAK). In addition, another 10% of the RSA measurements was performed twice by IB (no differences were detected).

To assess the image quality, RSA images were acquired with all the combinations of roentgen settings, including standard settings with and without external tube filtering.

**Figure 2.**
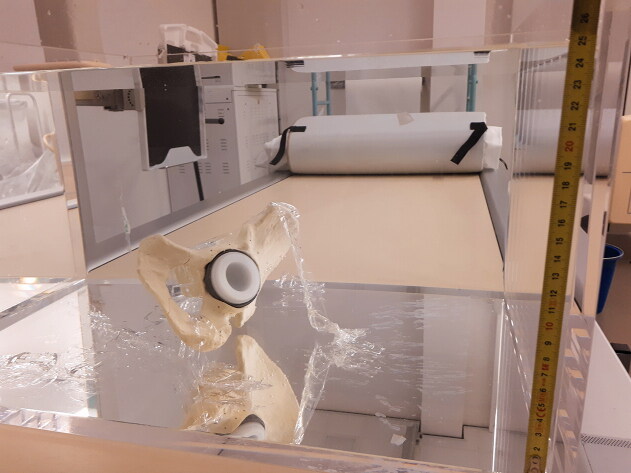
Sawbone of the hemipelvis placed in the Perspex box filled with 24.8 cm vertical water column.

**Figure 3.**
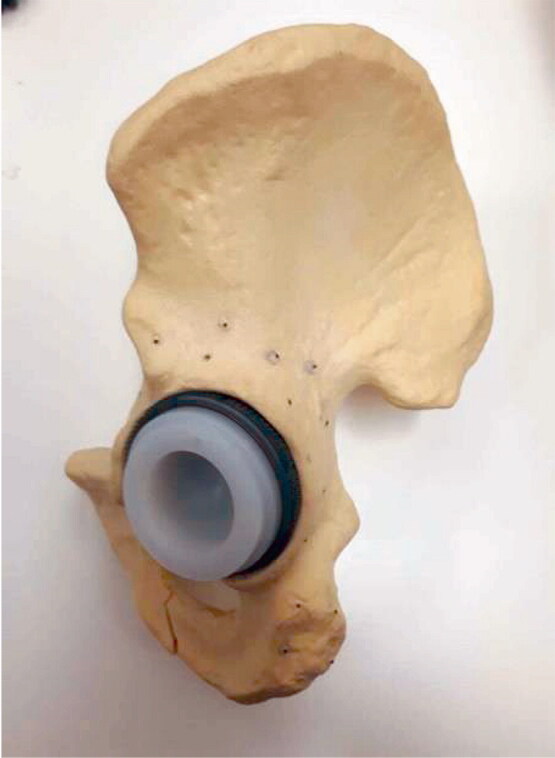
Phantom: sawbone of the hemipelvis, with the Allofit acetabular cup with a polyethylene liner (size 54 cm diameter) with tantalum markers attached around the acetabular bone.

To mimic a standard adult patient a phantom was placed in a Perspex box with walls of 12 mm filled with 24.8 cm water (Figure 2) (Slade-Schaaphok 2016). Perspex has almost the same density as human tissue and therefore the backscatter of the Perspex is similar to backscatter of human tissue (Slade-Schaaphok 2016). Furthermore, water is a good approximation of soft tissue of a human body (Sandborg 1990, Slade-Schaaphok 2016). The phantom model consisted of an Allofit Acetabular Cup with a highly crosslinked polyethylene liner (titanium outershell, size 54 mm, Zimmer Biomet, Warsaw, IN, USA) surgically placed in a hemipelvic sawbone (Sawbones, Vashon Island, WA, USA). 18 tantalum markers (1.0 mm diameter) were attached to the acetabulum (Figure 3), part cranially of the cup, part in the ischium bone. In between the RSA acquisitions the phantom and tube positions were left unchanged.

Acceptable image quality was defined as an image suitable for analysis with model-based RSA (version 4.2014, RSA*core*, department of Orthopedic Surgery, LUMC, Leiden, the Netherlands). Default model-based RSA settings require at least 6 non-collinear fiducial markers and 4 control markers to be correctly detected in each X-ray image in order to analyze an RSA radiograph (Kaptein et al. 2003). Furthermore, contour difference should be below 0.2 for optimal pose estimation of the CAD model, as defined by a performed phantom study of the model (mean contour difference of 0.2). For migration calculation (translation and rotation) at least 3 3D bone markers are required which need to meet the ISO standard (mean error of rigid body matching < 0.35 and condition number < 150 ms-1) (ISO 16087:2013).

For all different RSA radiographs the number of automatically detected calibration cage markers with Hough threshold of 16, the number of automatically detected markers attached to the pelvic sawbone, and the length of the automatically detected contour of the cup were scored. The longest contour detected within the region of contour detection around the acetabular cup projection, set by the analyst, was automatically selected by the software. Other contours that were considered by the analyst to be part of the acetabular cup contour were manually selected. Contours were not cut into smaller pieces.

### Funding and potential conflicts of interest

The authors declare no conflicts of interest condition number.

## ^Results^

### ^Effective dose^

Images with standard roentgen settings with no external tube filtration, 0.1 Cu + 1 Al and 0.2 Cu + 1 Al external tube filtration, have acceptable image quality. The lowest calculated ED was 0.044 mSv, on standard settings with an external tube filter of 0.2 Cu + 1 Al (Table 2). The maximum calculated ED was 0.094 mSv with standard settings and without external tube filtration.

**Table 2.** ED (mSv) with standard roentgen settings (medio-lateral 73 kV, 25 mAs; latero-medial 90 kV, 12.5 mAs, no external filtration)

Item	No filtration	2 mm Al	0.1 mm Cu+ 1 mm Al	0.2 mm Cu+ 1 mm Al
ED (mSv)	0.094	0.072 ^a^	0.061	0.044

**^a^** Setting that result in poor image quality; other settings resulted in acceptable image quality.

### ^Optimal roentgen settings^

Table 3 shows the results on ED and image quality of all tested combinations of roentgen settings (identical for both tubes). The lowest ED with acceptable image quality was 0.043 mSv with a roentgen technique of 90 kV, 12.5 mAs, and 0.2 Cu + 1 Al. The highest ED with acceptable image quality was 0.223 mSv with a roentgen technique of 102 kV, 25 mAs, and without external tube filtration. Between the lowest and highest ED with acceptable image quality is a difference of 0.18 mSv.

**Table 3.** ED (mSv) with various, but identical for both tubes, roentgen settings

Filtration/Voltage	8 mAs	12.5 mAs	16 mAs	25 mAs
0 mm Al				
77 kV	0.033 **^a^**	0.052 **^a^**	0.066	0.104 **^a^**
85 kV	0.044 **^a^**	0.069	0.088	0.138
90 kV	0.051 **^a^**	0.080 **^a^**	0.103 **^a^**	0.159
102 kV	0.071 **^a^**	0.111	0.143	0.223
2 mm Al				
77 kV	0.026 **^a^**	0.040 **^a^**	0.051 **^a^**	0.081
85 kV	0.035 **^a^**	0.054 **^a^**	0.069	0.108 **^a^**
90 kV	0.041 **^a^**	0.064 **^a^**	0.081	0.128
102 kV	0.057 **^a^**	0.089	0.114	0.179
0.1 mm Cu + 1 mm Al				
77 kV	0.022 **^a^**	0.034 **^a^**	0.044	0.069
85 kV	0.030 **^a^**	0.047 **^a^**	0.060	0.094
90 kV	0.036 **^a^**	0.056 **^a^**	0.071	0.112
102 kV	0.051 **^a^**	0.079	0.102 **^a^**	0.159
0.2 mm Cu + 1 mm Al				
77 kV	0.016 **^a^**	0.025 **^a^**	0.032 **^a^**	0.051 **^a^**
85 kV	0.023 **^a^**	0.036 **^a^**	0.046 **^a^**	0.072 **^a^**
90 kV	0.027 **^a^**	0.043	0.055	0.087
102 kV	0.040 **^a^**	0.063	0.080 **^a^**	0.125

**^a^** Setting that result in poor image quality; other settings resulted in acceptable image quality.

Tables 4 and 5 (see Supplementary data) provide the results of the ED measurements and the different roentgen settings on the RSA parameters used to determine image quality for RSA analysis.

## ^Discussion^

In this study we evaluated the ED of an RSA radiograph with standard roentgen settings in a hip phantom model using DR roentgen systems. Furthermore, we determined the optimal roentgen settings to acquire RSA radiographs in a hip phantom model that are acceptable for analysis while minimizing the radiation dose. We hypothesized that with the introduction of DR roentgen systems, the ED of hip RSA acquisitions could be reduced compared with CR roentgen systems while maintaining acceptable image quality for RSA analysis. In this study the RSA set-up consists of a fixed roentgen tube and a mobile roentgen tube. Several RSA research sites use a high-end system with 2 fixed tubes. It is assumed that this can result in a lower ED than measured in our study, because of better internal tube filtration and more powerful generators to generate higher roentgen techniques.

Based on the standard roentgen settings recommended by Valstar (2001) the ED of a single RSA radiograph of the hip using 2 DR systems was 0.094 mSv. This is lower compared with the reported ED of 0.150 mSv by Teeuwisse et al. (1998), who used the same settings in combination with CR systems. To our knowledge there is only 1 other publication that reports the ED of hip RSA radiographs in a phantom study, although image quality was not objectified in that study (Brodén et al. 2016). Furthermore, we used external tube filters to lower the ED, which has not been reported in any other RSA study so far.

Our results show that the optimal roentgen setting, a combination of the standard roentgen settings recommended by Valstar in combination with 0.2 Cu + 1 Al external tube filter, resulted in an ED of 0.043 mSv. However, this ED is based on imaging a standard patient. When patients have larger BMI, the roentgen technique should be adapted, resulting in a higher ED as larger kV and mAs are necessary. It is not expected that the roentgen technique resulting in the highest calculated ED (0.223 mSv) measured in this study is necessary for the THA patient with a larger BMI compared with the standard patient, in order to acquire images suitable for RSA analysis. CT-based RSA of the hip looks promising, with a calculated ED of 0.33 mSv for an experimental hip study (Brodén et al. 2016) and 0.2–2.3 mSv for a clinical hip study (Brodén et al. 2020). However, the range in radiation dose in this clinical study is quite wide. Though CT-based RSA could have advantages over RSA, further optimization of CT protocols is necessary to reduce the radiation dose in order to achieve acceptable radiation dose for patients in a long-term follow-up migration study.

### ^Clinical implications^

A standard RSA study typically consists of 6 RSA radiographs: 5 follow-up moments and a double examination. With optimal roentgen settings this results in a cumulative ED of approximately 0.26 mSv. RSA studies mostly fall into the category of increasing knowledge leading to a health benefit for the population, which is classified as a category IIa study. The acceptable cumulative ED for category IIa studies, according to EU guideline ‘Radiation Protection 99’, is 0.1–1.0 mSv (European Commission 1998). When adults over 50 years of age are participating in a category IIa study, the thresholds can be increased 5- to 10-fold, resulting in minimum thresholds of 0.5–5.0 mSv (ICRP 2007).

Our results show that the cumulative ED for a standard patient in a hip RSA study is far below the upper threshold applicable for this kind of study when the optimal settings are used for DR roentgen systems. Even using the highest calculated ED in this study for a single RSA radiograph, the cumulative ED is approximately 1.4 mSv. Adjustment of roentgen settings for patients with a higher BMI compared with the standard patient is thus unlikely to result in an ED above the 5.0 mSv.

### ^Limitations^

Our study has several limitations. To determine the ED we used an Alderson phantom, which is based on a standard patient with a BMI of 25.4. Therefore, the assessment of image quality was also performed mimicking a standard patient. Arthroplasty patients in our general RSA hip studies have a BMI of 28.1. Due to more soft tissue around the hip joint more radiation is necessary to obtain an acceptable roentgen image and, hence, these patients receive a higher ED. However, even without considering the age factor, which is usually over 50, adding additional kV and mAs will not result in unacceptable ED. For each individual patient the radiology assistant might change the roentgen settings to acquire acceptable-quality images. For some individual patients this results in higher ED compared with the results presented in this paper. It is important, however, to acquire acceptable-quality images suitable for analysis. In that perspective it is probably better to over-compensate the roentgen settings for patients with more soft tissue, than to be cautious with the radiation applied and then have to retake the RSA radiograph with higher settings due to poor image quality.

The calculated ED is the sum of the ED from the PCXMC software for each tube using the measured AD. Though this is an overestimation of the real ED, this calculated ED is lower than the known values in the literature or calculated using DoseAreaProduct calculations.

Another limitation of our study is the use of a hip phantom model. We used a hip phantom model because the roentgen settings, and therefore the ED, in a hip RSA study are higher compared with the settings in other joints in the extremities and because of the nature of the irradiated tissue. Though ED in the shoulder and the spine is probably higher, we opted to use the hip model as this is, together with the knee joint, the most frequently studied joint in RSA research. For spine and shoulder joints, the results of our study cannot be taken as an indication, but for other joints in the extremities we can confidently say that the ED for RSA acquisitions is below that of the hip. Normal DR of these joints has a lower ED than the hip (RIVM 2011).

In this study we have used Philips X-ray DR systems in combination with Skyplate detectors, a uniplanar carbon cage and a metal backed acetabular cup. There are, however, many different combinations of roentgen systems, RSA cages, and prostheses available. All the aforementioned factors will likely have an effect on the necessary roentgen settings and the ED for the patient. However, we do not expect that the variability of these parameters will results in an increase in the ED above the acceptable threshold of 1.0 mSv for patients under 50 years of ages (ICRP 2007).

Regarding image quality, we have used the default model-based RSA software settings with automatic marker detection and labelling option active. Based on the experience with the hardware and the visual evaluation of the images by the RSA analysts, the Hough threshold for marker detection was decreased. This resulted in a combination of roentgen settings, image quality, and detection settings that can be used for analysis without manually adjusting marker projections and contours. Experienced RSA analysts will be able to use even poorer image quality; however, the analysis of these images will require more time and migration results might be more sensitive to image noise.

The applied combination of roentgen settings can be optimized even further and could be made specific for each joint in the extremities and for different kind of prostheses or roentgen hardware used. We believe however, that the optimization for these possibilities will result in even lower ED and this will not have any implications for the use of RSA in standard RSA studies considering the applicable radiation threshold for research purposes.

## ^Conclusion^

The lowest calculated ED of an RSA radiograph with standard roentgen settings and an external tube filter of 0.2 Cu + 1 mm Al was 0.044 mSv. This is more than 0.1 mSv lower than the given 0.150 mSv as stated by Teeuwisse et al. (1998).

With modern DR equipment, the roentgen technique for both tubes of 90 kV, 12.5 mAs, and 0.2 mm Cu + 1 mm Al gave the optimal result: an ED of 0.043 mSv and good image quality.

The accumulated ED for a patient in a 2-year clinical hip RSA study with 5 FU moments and a double acquisition is below the acceptable threshold of 1.0 mSv provided by the EU radiation guideline for studies increasing knowledge for the general health of the population.

The double examination in a regular RSA study is essential to determine the clinical precision of RSA (ISO 16087:2013). Though it is an additional RSA acquisition, this does not result in exceeding the threshold for ED of 1.0 mSv (ICRP 2007). As a result the additional radiation dose from the double examination does not have to be a reason for Medical Ethics Committees to prohibit the acquisition of the double examination (Valstar et al. 2005).

## Supplementary Material

Supplemental Material

## ^Supplementary data^

Tables 4 and 5 are available as supplementary data in the online version of this article, http://dx.doi.org/10.1080/17453674. 2020.1767443
