# Supplementary material for: Effective radiation dose in radiostereometric analysis of the hip
Source: Acta Orthop. 2020 May 26;91(4):1–395. doi: 10.1080/17453674.2020.1767443 (PMC8023876; doi:10.1080/17453674.2020.1767443)
Supplement: Supplemental Material [file IORT_A_1767443_SM7929.pdf]

# Supplementary data

**Table 4. Effective radiation dose (ED) in mSv, calibration information and image quality score of various roentgen settings without and with external tube filtration**

| kV ml + lm | mAs ml + lm | A | B | C | D | E | F | G | H | I | J | K | L | M | N | O | P | Q |
|---|---|---|---|---|---|---|---|---|---|---|---|---|---|---|---|---|---|---|
| No filtration | | | | | | | | | | | | | | | | | | |
| 73 + 90 | 25 + 12.5 | 0.094 | 14 | 0.036 | 8 | 0.514 | 14 | 0.026 | 8 | 0.188 | 11 | 10 | 9 | 0.010 | 1,410 | 1,747 | 0.21 | 1 |
| 125 | 5 + 6 | 0.074 | 11 | 0.034 | 9 | 0.344 | 23 | 0.039 | 12 | 0.207 | 11 | 9 | 7 | 0.052 | 792 | 1,517 | 0.24 | 1 |
| 125 | 6 + 5 | 0.089 | 0 | N/A | 0 | N/A | 23 | 0.039 | 12 | 0.190 | 10 | 10 | N/A | N/A | 659 | 1,791 | N/A | 2 |
| 2 mm Al | | | | | | | | | | | | | | | | | | |
| 73 + 90 | 25 + 12.5 | 0.072 | 0 | N/A | 0 | N/A | 18 | 0.028 | 11 | 0.239 | 12 | 8 | N/A | N/A | 1,117 | 1,699 | N/A | 2 |
| 125 | 5 + 6 | 0.062 | 12 | 0.030 | 10 | 0.256 | 16 | 0.048 | 12 | 0.195 | 11 | 10 | 7 | 0.019 | 861 | 1,459 | 0.25 | 1 |
| 125 | 6 + 5 | 0.072 | 0 | N/A | 0 | N/A | 23 | 0.030 | 12 | 0.138 | 9 | 9 | N/A | N/A | 513 | 1,589 | N/A | 2 |
| 0.1 mm CU + 1 mm Al | | | | | | | | | | | | | | | | | | |
| 73 + 90 | 25 + 12.5 | 0.061 | 18 | 0.030 | 9 | 0.293 | 18 | 0.044 | 9 | 0.246 | 15 | 8 | 9 | 0.013 | 1,270 | 1,788 | 0.21 | 1 |
| 125 | 5 + 6 | 0.055 | 10 | 0.018 | 6 | 0.372 | 21 | 0.067 | 10 | 0.226 | 11 | 10 | 8 | 0.034 | 633 | 1,521 | 0.21 | 1 |
| 125 | 6 + 5 | 0.064 | 0 | N/A | 0 | N/A | 22 | 0.032 | 12 | 0.208 | 4 | 10 | N/A | N/A | 647 | 1,550 | N/A | 2 |
| 0.2 mm CU + 1 mm Al | | | | | | | | | | | | | | | | | | |
| 73 + 90 | 25 + 12.5 | 0.044 | 8 | 0.031 | 15 | 0.577 | 11 | 0.028 | 9 | 0.424 | 9 | 7 | 7 | 0.085 | 787 | 1,446 | 0.15 | 1 |
| 125 | 5 + 6 | 0.044 | 10 | 0.054 | 4 | 58.697 | 20 | 0.038 | 11 | 0.258 | 9 | 9 | N/A | N/A | 670 | 1,200 | N/A | 2 |
| 125 | 6 + 5 | 0.052 | 13 | 0.033 | 0 | N/A | 20 | 0.025 | 11 | 0.105 | 6 | 10 | N/A | N/A | 465 | 1,502 | N/A | 2 |

kV = kilovoltage, ml = medio-lateral, lm = latero-medial, mAs = milliampere-seconds, Cu = copper, Al = aluminum
A. Effective radiation dose (ED) in millisievert (mSv)
B. Number of fiducial markers left image
C. Error of fiducial markers left image
D. Number of control markers left image
E. Error of control markers left image
F. Number of fiducial markers right image
G. Error of fiducial markers right image
H. Number of control markers right image
I. Error of control markers right image,
J. Number of acetabulum bone markers left image
K. Number of acetabulum bone markers right image
L. Number of 3D markers
M. Crossing line distance
N. Contour detection left image
O. Contour detection right image
P. Difference value
Q. Image quality is defined as good image quality with analyzable image (1) or poor image quality with not analyzable image (2).

**Table 5. Effective radiation dose (ED) in mSv, calibration information and image quality score of various roentgen settings without and with external tube filtration**

| kV | mAs | A | B | C | D | E | F | G | H | I | J | K | L | M | N | O | P | Q |
|---|---|---|---|---|---|---|---|---|---|---|---|---|---|---|---|---|---|---|
| No filtration | | | | | | | | | | | | | | | | | | |
| 77 | 8 | 0.033 | 0 | N/A | 0 | N/A | 0 | N/A | 0 | N/A | 5 | 5 | N/A | N/A | 825 | 1,368 | N/A | 2 |
| 77 | 12.5 | 0.052 | 0 | N/A | 0 | N/A | 18 | 0.026 | 11 | 0.286 | 10 | 8 | N/A | N/A | 1,194 | 1,382 | N/A | 2 |
| 77 | 16 | 0.066 | 9 | 0.020 | 8 | 0.341 | 23 | 0.035 | 11 | 0.291 | 12 | 11 | 13 | 0.063 | 1,276 | 1,786 | 0.16 | 1 |
| 77 | 25 | 0.104 | 10 | 0.018 | 4 | 52.16 | 24 | 0.027 | 12 | 0.195 | 10 | 10 | N/A | N/A | 1358 | 1,758 | N/A | 2 |
| 85 | 8 | 0.044 | 0 | N/A | 0 | N/A | 15 | 0.039 | 10 | 0.348 | 8 | 9 | N/A | N/A | 864 | 1,540 | N/A | 2 |
| 85 | 12.5 | 0.069 | 20 | 0.034 | 9 | 0.284 | 21 | 0.032 | 11 | 0.257 | 12 | 10 | 10 | 0.031 | 1,238 | 1,696 | 0.19 | 1 |
| 85 | 16 | 0.088 | 14 | 0.024 | 11 | 0.236 | 23 | 0.021 | 11 | 0.145 | 13 | 10 | 12 | 0.023 | 1,491 | 1,728 | 0.21 | 1 |
| 85 | 25 | 0.138 | 11 | 0.042 | 11 | 0.395 | 23 | 0.021 | 11 | 0.135 | 14 | 10 | 11 | 0.031 | 1,136 | 1,789 | 0.18 | 1 |
| 90 | 8 | 0.051 | 0 | N/A | 0 | N/A | 22 | 0.028 | 12 | 0.380 | 9 | 10 | N/A | N/A | 945 | 1,407 | N/A | 2 |
| 90 | 12.5 | 0.080 | 0 | N/A | 0 | N/A | 22 | 0.025 | 11 | 0.175 | 12 | 10 | N/A | N/A | 921 | 1,537 | N/A | 2 |
| 90 | 16 | 0.103 | 13 | 0.036 | 0 | N/A | 23 | 0.027 | 11 | 0.239 | 11 | 10 | N/A | N/A | 1,078 | 1,785 | N/A | 2 |
| 90 | 25 | 0.159 | 17 | 0.027 | 8 | 0.195 | 24 | 0.032 | 12 | 0.181 | 12 | 11 | 9 | 0.043 | 1,028 | 1,815 | 0.16 | 1 |
| 102 | 8 | 0.071 | 0 | N/A | 0 | N/A | 21 | 0.026 | 12 | 0.187 | 10 | 10 | N/A | N/A | 926 | 1,705 | N/A | 2 |
| 102 | 12.5 | 0.111 | 16 | 0.034 | 10 | 0.483 | 23 | 0.024 | 11 | 0.258 | 11 | 10 | 9 | 0.048 | 819 | 1,792 | 0.18 | 1 |
| 102 | 16 | 0.143 | 16 | 0.054 | 11 | 0.462 | 23 | 0.041 | 12 | 0.154 | 10 | 12 | 11 | 0.042 | 1,067 | 1,732 | 0.2 | 1 |
| 102 | 25 | 0.223 | 17 | 0.032 | 12 | 0.358 | 24 | 0.037 | 12 | 0.176 | 13 | 11 | 13 | 0.042 | 996 | 1,860 | 0.19 | 1 |
| 2 mm Al | | | | | | | | | | | | | | | | | | |
| 77 | 8 | 0.026 | 0 | N/A | 0 | N/A | 0 | N/A | 0 | N/A | 0 | 4 | N/A | N/A | 641 | 1.445 | N/A | 2 |
| 77 | 12.5 | 0.04 | 0 | N/A | 0 | N/A | 14 | 0.024 | 7 | 14.229 | 6 | 7 | N/A | N/A | 746 | 1.473 | N/A | 2 |
| 77 | 16 | 0.051 | 0 | N/A | 0 | N/A | 20 | 0.087 | 11 | 0.215 | 13 | 8 | N/A | N/A | 1.225 | 1.568 | N/A | 2 |
| 77 | 25 | 0.081 | 8 | 0.009 | 6 | 0.359 | 22 | 0.028 | 12 | 0.224 | 11 | 10 | 8 | 0.054 | 1.034 | 1.832 | 0.17 | 1 |
| 85 | 8 | 0.035 | 0 | N/A | 0 | N/A | 16 | 0.029 | 11 | 0.583 | 11 | 9 | N/A | N/A | 912 | 1.310 | N/A | 2 |
| 85 | 12.5 | 0.054 | 0 | N/A | 0 | N/A | 21 | 0.030 | 12 | 0.187 | 8 | 10 | N/A | N/A | 772 | 1.725 | N/A | 2 |
| 85 | 16 | 0.069 | 10 | 0.021 | 7 | 0.379 | 19 | 0.026 | 12 | 0.216 | 13 | 10 | 11 | 0.029 | 1.112 | 1.796 | 0.18 | 1 |
| 85 | 25 | 0.108 | 18 | 0.029 | 0 | N/A | 22 | 0.026 | 11 | 0.187 | 12 | 10 | N/A | N/A | 932 | 1.749 | N/A | 2 |
| 90 | 8 | 0.041 | 0 | N/A | 0 | N/A | 23 | 0.050 | 12 | 0.230 | 9 | 9 | N/A | N/A | 853 | 1.560 | N/A | 2 |
| 90 | 12.5 | 0.064 | 0 | N/A | 0 | N/A | 22 | 0.031 | 12 | 0.192 | 7 | 10 | N/A | N/A | 1.010 | 1.603 | N/A | 2 |
| 90 | 16 | 0.081 | 13 | 0.035 | 7 | 0.285 | 23 | 0.021 | 12 | 0.179 | 12 | 9 | 9 | 0.043 | 1.137 | 1.831 | 0.18 | 1 |
| 90 | 25 | 0.127 | 16 | 0.036 | 11 | 0.481 | 22 | 0.028 | 12 | 0.181 | 12 | 12 | 12 | 0.031 | 1.020 | 1.751 | 0.19 | 1 |
| 102 | 8 | 0.057 | 0 | N/A | 0 | N/A | 22 | 0.028 | 12 | 0.282 | 8 | 10 | N/A | N/A | 845 | 1.576 | N/A | 2 |
| 102 | 12.5 | 0.089 | 13 | 0.043 | 7 | 0.296 | 24 | 0.028 | 12 | 0.157 | 11 | 11 | 10 | 0.035 | 973 | 1.723 | 0.18 | 1 |
| 102 | 16 | 0.114 | 18 | 0.032 | 8 | 0.337 | 24 | 0.032 | 11 | 0.182 | 12 | 10 | 10 | 0.036 | 819 | 1.740 | 0.17 | 1 |
| 102 | 25 | 0.179 | 19 | 0.037 | 12 | 0.280 | 24 | 0.025 | 12 | 0.238 | 13 | 9 | 10 | 0.017 | 854 | 1.802 | 0.17 | 1 |
| 0.1 mm Cu + 1 mm Al | | | | | | | | | | | | | | | | | | |
| 77 | 8 | 0.022 | 0 | N/A | 0 | N/A | 0 | N/A | 0 | N/A | 3 | 4 | N/A | N/A | 602 | 1,324 | N/A | 2 |
| 77 | 12.5 | 0.034 | 0 | N/A | 0 | N/A | 10 | 0.025 | 9 | 0.250 | 8 | 8 | N/A | N/A | 873 | 1,536 | N/A | 2 |
| 77 | 16 | 0.044 | 0 | N/A | 0 | N/A | 14 | 0.028 | 9 | 0.266 | 10 | 7 | N/A | N/A | 1,147 | 1,832 | N/A | 2 |
| 77 | 25 | 0.069 | 11 | 0.032 | 5 | 0.464 | 23 | 0.027 | 12 | 0.204 | 10 | 10 | 10 | 0.04 | 1,280 | 1,777 | 0.17 | 1 |
| 85 | 8 | 0.030 | 0 | N/A | 0 | N/A | 12 | 0.032 | 11 | 0.244 | 6 | 10 | 0 | N/A | 842 | 1,681 | N/A | 2 |
| 85 | 12.5 | 0.014 | 0 | N/A | 0 | N/A | 20 | 0.024 | 11 | 0.192 | 8 | 8 | N/A | N/A | 905 | 1,723 | N/A | 2 |
| 85 | 16 | 0.060 | 16 | 0.033 | 11 | 0.329 | 22 | 0.033 | 12 | 0.149 | 13 | 9 | 11 | 0.026 | 1,530 | 1,753 | 0.20 | 1 |
| 85 | 25 | 0.094 | 12 | 0.026 | 9 | 0.286 | 23 | 0.030 | 12 | 0.146 | 11 | 10 | 9 | 0.02 | 931 | 1,662 | 0.18 | 1 |
| 90 | 8 | 0.036 | 0 | N/A | 0 | N/A | 18 | 0.035 | 10 | 0.218 | 6 | 7 | N/A | N/A | 767 | 1,393 | N/A | 2 |
| 90 | 12.5 | 0.056 | 0 | N/A | 0 | N/A | 23 | 0.024 | 12 | 0.209 | 11 | 10 | N/A | N/A | 1,366 | 1,358 | N/A | 2 |
| 90 | 16 | 0.071 | 16 | 0.026 | 11 | 0.65 | 22 | 0.028 | 12 | 0.298 | 13 | 10 | 10 | 0.017 | 1,337 | 1,747 | 0.20 | 1 |
| 90 | 25 | 0.112 | 11 | 0.039 | 8 | 0.351 | 23 | 0.055 | 12 | 0.172 | 8 | 10 | 7 | 0.024 | 1,006 | 1,819 | 0.21 | 1 |
| 102 | 8 | 0.051 | 0 | N/A | 0 | N/A | 23 | 0.038 | 12 | 0.257 | 7 | 11 | N/A | N/A | 753 | 1,335 | N/A | 2 |
| 102 | 12.5 | 0.079 | 9 | 0.025 | 11 | 0.407 | 23 | 0.024 | 12 | 0.222 | 8 | 8 | 5 | 0.039 | 870 | 1,687 | 0.19 | 1 |
| 102 | 16 | 0.102 | 16 | 0.044 | 5 | 14.459 | 24 | 0.023 | 12 | 0.171 | 12 | 10 | N/A | N/A | 824 | 1,590 | N/A | 2 |
| 102 | 25 | 0.159 | 22 | 0.036 | 12 | 0.288 | 24 | 0.031 | 12 | 0.210 | 14 | 10 | 12 | 0.037 | 1,238 | 1,672 | 0.20 | 1 |
| 0.2 mm Cu + 1 mm Al | | | | | | | | | | | | | | | | | | |
| 77 | 8 | 0.016 | 0 | N/A | 0 | N/A | 0 | N/A | 0 | N/A | 0 | 2 | N/A | N/A | 508 | 1,229 | N/A | 2 |
| 77 | 12.5 | 0.025 | 0 | N/A | 0 | N/A | 0 | N/A | 0 | N/A | 6 | 8 | N/A | N/A | 919 | 1,367 | N/A | 2 |
| 77 | 16 | 0.032 | 0 | N/A | 0 | N/A | 17 | 0.040 | 9 | 0.392 | 5 | 8 | N/A | N/A | 917 | 1,341 | N/A | 2 |
| 77 | 25 | 0.051 | 0 | N/A | 0 | N/A | 20 | 0.069 | 11 | 0.222 | 9 | 10 | N/A | N/A | 1,338 | 1,573 | N/A | 2 |
| 85 | 8 | 0.023 | 0 | N/A | 0 | N/A | 10 | 0.024 | 7 | 14.522 | 8 | 8 | N/A | N/A | 746 | 1,134 | N/A | 2 |
| 85 | 12.5 | 0.036 | 0 | N/A | 0 | N/A | 20 | 0.041 | 8 | 0.166 | 10 | 10 | N/A | N/A | 980 | 1,534 | N/A | 2 |
| 85 | 16 | 0.046 | 0 | N/A | 0 | N/A | 22 | 0.030 | 12 | 0.310 | 9 | 10 | N/A | N/A | 704 | 1,596 | N/A | 2 |
| 85 | 25 | 0.072 | 16 | 0.049 | 8 | 0.443 | 23 | 0.022 | 12 | 0.224 | 11 | 11 | 10 | 0.06 | 1,145 | 1,808 | 0.21 | 1 |
| 90 | 8 | 0.027 | 0 | N/A | 0 | N/A | 19 | 0.033 | 11 | 0.209 | 7 | 9 | N/A | N/A | 823 | 1,388 | N/A | 2 |
| 90 | 12.5 | 0.043 | 8 | 0.023 | 5 | 0.640 | 22 | 0.025 | 12 | 0.254 | 8 | 10 | 6 | 0.026 | 850 | 1,583 | 0.17 | 1 |
| 90 | 16 | 0.055 | 10 | 0.031 | 7 | 0.385 | 22 | 0.034 | 12 | 0.220 | 10 | 10 | 7 | 0.059 | 946 | 1,692 | 0.20 | 1 |
| 90 | 25 | 0.087 | 15 | 0.033 | 10 | 0.38 | 22 | 0.029 | 12 | 0.147 | 14 | 11 | 12 | 0.057 | 795 | 1,732 | 0.16 | 1 |
| 102 | 8 | 0.040 | 0 | N/A | 0 | N/A | 21 | 0.037 | 12 | 0.233 | 11 | 9 | N/A | N/A | 693 | 1,389 | N/A | 2 |
| 102 | 12.5 | 0.063 | 12 | 0.033 | 6 | 0.532 | 24 | 0.024 | 12 | 0.198 | 9 | 9 | 6 | 0.042 | 630 | 1,596 | 0.18 | 1 |
| 102 | 16 | 0.080 | 0 | N/A | 0 | N/A | 24 | 0.041 | 12 | 0.226 | 10 | 10 | N/A | N/A | 623 | 1,601 | N/A | 2 |
| 102 | 25 | 0.125 | 18 | 0.017 | 11 | 0.292 | 24 | 0.038 | 12 | 0.160 | 12 | 9 | 9 | 0.043 | 836 | 1,661 | 0.16 | 1 |

For footnotes, see Table 4.
